# Supplementary material for: NMR and SEM fractal dimensions explore shale pore structure taking the Upper Paleozoic shale in Ordos Basin as an example
Source: PLoS One. 2025 May 29;20(5):e0323968. doi: 10.1371/journal.pone.0323968 (PMC12122040; doi:10.1371/journal.pone.0323968)
Supplement: Attachment — Submitted filename: PONE-D-24-42271 review.pdf [file pone.0323968.s002.pdf]

In this paper, authors proposed to examine the pore structure and fractal characteristics of shale in the Upper Paleozoic Shanxi Formation of the Ordos Basin. The study analyses the relationship between the NMR fractal dimension and factors such as mineral composition, geochemical parameters, and physical properties, utilizing SEM imaging and artificial intelligence techniques.

The writing quality of the paper is of a moderate level so I recommend authors to improve the general quality of the writing based on the comments hereafter especially for the introduction which is quite weak.

Technically, results in this study are interesting but I do not see novelty in the paper and I urge authors to emphasize the novelty in the approach as I could not see it clearly. Moreover, I have several questions and suggestions to improve the paper content and quality as at this stage.

Introduction: it should be rewritten significantly based on the following comments. Also authors, should emphasize in this section about the novelty of the approach compared to previous studies.

Introduction:

Shale reservoir **has** →Shale reservoir**s have**

Predecessors→ Previous studies examined

Please replace all shale reservoir → **shale reservoirs**

This is unclear you need to explain: Based on the evaluation parameters such as area porosity and coefficient of variation.

At present, the fractal dimension is mainly obtained by SEM image analysis → This statement is not true and should be corrected indeed several research studies used FIB-SEM, 3D X-Ray Micro-Computed. Please see the list of papers that you should refer to in your paper.

- Sulieman H, Jouini MS, Alsuwaidi M, Al-Shalabi EW, Al Jallad OA **(2024)** Multiscale investigation of pore structure heterogeneity in carbonate rocks using digital imaging and SCAL measurements: A case study from Upper Jurassic limestones, Abu Dhabi, UAE. PLoS ONE 19(2): e0295192. https://doi.org/10.1371/journal.pone.0295192
- Jouini, M.S., Alabere, A.O., Alsuwaidi, M. et al. Experimental and digital investigations of heterogeneity in lower cretaceous carbonate reservoir using fractal and multifractal concepts, **(2023).** *Scientific Reports* 13, 20306 https://doi.org/10.1038/s41598-023-47681-w

SEM image analysis is a means to directly observe pore morphology and Structure → SEM image analysis provides a direct method for observing pore morphology and structure.

NMR experiment has the characteristics of non-destructive, fast and accurate → NMR experiments are non-destructive, fast, and highly accurate.

Section 3.2 Authors stated that they open the Weka Segmentation artificial intelligence tool and use it to improve the image segmentation results. However they did not specify which AI method was used what is its sensitivity advantages and limitations.

Section 3.5 Traditionally, the usual formula used for Dathe's formula is based on box-counting technique to compute the fractal dimension D_f:

$$D_f = \lim_{\epsilon \to 0} \frac{\log(N(\epsilon))}{\log(1/\epsilon)}$$

- N(ϵ) is the number of boxes of side length ϵ that are needed to cover the structure and ϵ represents the box size.

The equation provided in (1) is not common so please provide references at least to know who proposed it and in which context it was used.

Section 4.3 Draw fractal curve -> please change the title

Section 5.1 Discussion The reasons may be that quartz pores are less developed and its contribution to reservoir space is weak rephrase to: The reasons may be that quartz pores are underdeveloped, contributing minimally to the reservoir space.

Several authors tried to investigate possible relationship between experimental fractal dimension (here NMR) and image fractal dimension (here SEM). Since you have all the results could you provide any comments regarding this?
