# Supplementary material for: NMR and SEM fractal dimensions explore shale pore structure taking the Upper Paleozoic shale in Ordos Basin as an example
Source: PLoS One. 2025 May 29;20(5):e0323968. doi: 10.1371/journal.pone.0323968 (PMC12122040; doi:10.1371/journal.pone.0323968)
Supplement: Attachment — Submitted filename: PONE-D-24-42271_R1 review.pdf [file pone.0323968.s004.pdf]

In this paper, authors proposed to examine the pore structure and fractal characteristics of shale in the Upper Paleozoic Shanxi Formation of the Ordos Basin. The study analyses the relationship between the NMR fractal dimension and factors such as mineral composition, geochemical parameters, and physical properties, utilizing SEM imaging and artificial intelligence techniques.

Several of my comments stated in my first revision were ignored and left without response. Authors should either implement suggested improvements or reply why they refuse to implement them. They cannot ignore reviewers comments it is not acceptable. Also, the writing quality of the paper still need significant improvement.

Introduction:

The introduction is too short and does not highlight enough the work done in fractals to characterize rock properties. It need a significant re-writing.

- Authors did not highlighted the novelty in the approach as I requested in my first revision.
- **1 introduction -> Introduction**
- shale reservoir **->** Shale reservoir
- “At present, the fractal dimension is mainly obtained by SEM image analysis”

This statement is not true and should be corrected indeed several research studies used FIB-SEM, 3D X-Ray Micro-Computed. Please see the list of papers that you should refer to in your paper.

- Sulieman H, Jouini MS, Alsuwaidi M, Al-Shalabi EW, Al Jallad OA **(2024)** Multiscale investigation of pore structure heterogeneity in carbonate rocks using digital imaging and SCAL measurements: A case study from Upper Jurassic limestones, Abu Dhabi, UAE. PLoS ONE 19(2): e0295192. https://doi.org/10.1371/journal.pone.0295192
- Jouini, M.S., Alabere, A.O., Alsuwaidi, M. et al. Experimental and digital investigations of heterogeneity in lower cretaceous carbonate reservoir using fractal and multifractal concepts, **(2023).** *Scientific Reports* 13, 20306 https://doi.org/10.1038/s41598-023-47681-w

- **Section 4.2** Authors stated that they open the Weka Segmentation artificial intelligence tool and use it to improve the image segmentation results. However they did not specify which AI method was used what is its sensitivity advantages and limitations.

- **Section 4.3** Traditionally, the usual formula used for Dathe's formula is based on box-counting technique to compute the fractal dimension D_f:

$$D_f = \lim_{\epsilon \to 0} \frac{\log(N(\epsilon))}{\log(1/\epsilon)}$$

➢ $N(\epsilon)$ is the number of boxes of side length ϵ that are needed to cover the structure and ϵ represents the box size.

- The equation provided in (1) is not common so please provide references at least to know who proposed it and in which context it was used.

- Several authors tried to investigate possible relationship between experimental fractal dimension (here NMR) and image fractal dimension (here SEM). Since you have all the results could you provide any comments regarding this?
