# Supplementary material for: NMR and SEM fractal dimensions explore shale pore structure taking the Upper Paleozoic shale in Ordos Basin as an example
Source: PLoS One. 2025 May 29;20(5):e0323968. doi: 10.1371/journal.pone.0323968 (PMC12122040; doi:10.1371/journal.pone.0323968)
Supplement: Attachment — Submitted filename: Modify the description.docx [file pone.0323968.s005.docx]

Dear Editor:

These are the revisions made to the manuscript based on the review comments.

For Comment 1: The format of the article has been revised.

For Comment 2 (1): The model of the nuclear magnetic resonance (NMR) spectrometer and the magnetic field strength have been added. All samples were tested using the same instrument and under the same pressure conditions. Since the samples are shale, there is no information on cell nuclei; sample concentration; solvent used for dissolving the samples and whether solvent signal suppression was applied; reference standards and temperature, etc. (2) (3) The NMR testing of shale focuses on measuring the water and gas saturation after centrifugation, analyzing the development degree of shale pores through the difference in saturation before and after centrifugation, without detecting compound information. I will submit the NMR test report to the database. For Comment 3: The research content of this paper originates from my doctoral dissertation, so no permits were required. For Comment 4: The funders had no role in study design, data collection and analysis, decision to publish, or preparation of the manuscript. The research start-up funding provided economic support for me to conduct this part of the research at this institution. For Comment 5: Additional data has been supplemented and uploaded to PACE. The images were generated by software, which the PACE system does not accept.

Response to Reviewer 1's Comments:

Added maps showing the location of the study area, well positions, and profiles.

1. The advantage of FIB-SEM experiments lies in their capability for microfabrication of materials. SEM experiments are primarily used for observing surface morphology and compositional analysis of materials but do not possess microfabrication capabilities. This study mainly focuses on observing the pore structure characteristics of rock samples, thus SEM experimental analysis was adopted.
2. Provided a brief introduction to the method of smart pore recognition using ImageJ Weka Segmentation and described its advantages.
3. Revised the sentences that needed modification as suggested by the reviewer. We are very grateful for the valuable comments provided by the reviewer.
4. Made comprehensive revisions to the abstract of the paper.

Response to Reviewer 2's Comments:

1. Revised the format of the paper according to the publication standards.
2. Added regional geological maps.
3. Modified the content of the paper, removing redundant parts. Text corresponding to Figures 3(c) and (d) has been revised. Described the function of Formula 1. Revised Figure 5. Unified the scale of the y-axis for Figures 7 and 8. We are very grateful for the valuable comments provided by the reviewer.
