# Supplementary material for: NMR and SEM fractal dimensions explore shale pore structure taking the Upper Paleozoic shale in Ordos Basin as an example
Source: PLoS One. 2025 May 29;20(5):e0323968. doi: 10.1371/journal.pone.0323968 (PMC12122040; doi:10.1371/journal.pone.0323968)
Supplement: Attachment — Submitted filename: Response to Reviewers-2.docx [file pone.0323968.s006.docx]

To Reviewers：

1. The introduction of the paper was revised.

2. The abstract of the paper is revised and the innovations of this paper are highlighted

3. Reviewer's "1 introduction" and "shale reservoirs""At present, the fractal dimension is mainly obtained by SEM image analysis" have been revised.

4.Image J Weka Segmentation is an intelligent identification module in ImageJ software. It was originally used for cell identification in medicine, but used in this paper to identify the pore structure of shale.

5.The fractal dimension of SEM images adopts the perimeter-area method, which is used in "Fractal Method for Estimating Porosity","Calculation of Fractal Dimension of Soil","Fractal Dimension Measured from Area-Perimeter Relationship and Material Toughness" and other articles. Fractal dimension method is a method used to describe and analyze complex shapes and structures. Since this method can study the fractal dimension of soil, urban boundary dimension and other aspects, it can of course also be used to study the characteristics of shale pores.Dathe's The Surface Fractal Dimension of Soil-pore Interface as Measured by Image Analysis. The pore area and perimeter in the SEM image in this paper can be calculated by ImageJ software, so this method is used to calculate the fractal dimension of the pore.Therefore, this paper quotes the perimeter-area method used in Dathe's paper to calculate the pore fractal dimension in SEM images.
