# Supplementary material for: NMR and SEM fractal dimensions explore shale pore structure taking the Upper Paleozoic shale in Ordos Basin as an example
Source: PLoS One. 2025 May 29;20(5):e0323968. doi: 10.1371/journal.pone.0323968 (PMC12122040; doi:10.1371/journal.pone.0323968)
Supplement: Attachment — Submitted filename: Response to Reviewers-3.docx [file pone.0323968.s008.docx]

Dear reviewer：

Thank you for your constructive comments on my manuscript.We agree with the suggestions and will incorporate the recommended changes into the manuscript.

1. Based on Reviewer #1, after reading the references given by Reviewer, the statement "Authors stated: " Currently, there are three main methods to obtain the fractional "is revised to “Currently, the main methods to obtain the fractal dimension of shale pores include: SEM image analysis [14-15], nitrogen adsorption model [16], mercury porosimetry [17], thin slices [18], FIB-SEM[19] and multifractal theory based on 3D X-ray micro-computed tomography images [20], etc..” (see revised pages 2). References have been revised simultaneously(see revised pages 17).
2. The binary processing method is explained, and the default iteration method of Image J is adopted: The image is segmented into two parts by assuming a threshold T. The average values M1 and M2 are calculated for each part. Assuming that T '=(M1+M2) is not equal to T, then T=T' is made and then iterative continues until the two are equal(see revised pages 6-7).

We sincerely appreciate the time and effort invested by the reviewers in evaluating our manuscript. We look forward to any additional feedback or suggestions.

Yours sincerely
Zhao Keying, Zhang Zhanghua
7-Apr，2025
